# Supplementary material for: B Cells Induce Early-Onset Maternal Inflammation to Protect against LPS-Induced Fetal Rejection
Source: Int J Mol Sci. 2023 Nov 8;24(22):16091. doi: 10.3390/ijms242216091 (PMC10671511; doi:10.3390/ijms242216091)
Supplement: Supplementary file 1 [file ijms-24-16091-s001.zip › ijms-2580783-supplementary.pdf]

	WT PBS	WT LPS	$CD19^{-/-}$ PBS	$CD19^{-/-}$ LPS	$BMyD88^{-/-}$ PBS	$BMyD88^{-/-}$ LPS	μMT PBS	μMT LPS	*p*
chemokines									
IP-10	0.8	24.2	32.1	18.4	35.0	23.4	12.5	7.5	0.3345
LIX	0.0	2175	61.5	0	64.5	160.8	0	0*	0.0371
MDC	33.6*	427.6	40.8	276.3	38.0	311.1	58.5	70.2	0.0132
MIP-1α	0	91.1	65.8	114.8	116.2	323.2	57.1	35.4	0.0164
MIP-1β	58.0	68.5	89.2	0	83.4	64.5	60.9	19.7	0.3564
Eotaxin	14.3	77.2	78.0	131.3	125.9	75.5	48.1	75.0	0.2084
KC	0	180.9	0	141.0	158.0	154.6	0	348.7*	<0.0001
MCP-1	0*	133.1	0*	53.7	0*	132.0	0*	87.6	0.0003
MIP-3α	0	3.0	0	44.5	0	38.1	0	22.2	0.3123
RANTES	35.4	38.6	62.9	43.3	47.7	48.0	32.1	42.5	0.2054
TARC	0*	2707	51.8*	20.1*	23.0*	77.0*	0*	27.3*	0.0018
cytokines									
IL-6	0*	13835	0*	5497*	1.0*	5407*	0*	7402*	<0.0001
TNF-α	14.8*	392.3	17.7*	258.9	25.5*	212.8*	5.8*	203.2*	<0.0001
IL-2	0.8	1.8	0.8	2.7	4.4	1.2	1.2	6.2	0.4816
IL-4	2.2	0	5.4	0	7.4	0	3.2	10.2	0.1491
IL-10	20.6*	164.1	27.5*	20.9*	4.9*	36.9*	16.6*	5.1*	0.0086
IFN-γ	2.3	7.8	1.0	1.0	6.5	3.4	1.7	7.3	0.9049
IL-17A	3.5*	43.0	2.5*	22.5*	6.9*	10.0*	5.3*	10.0*	0.0006

Table S1: Concentrations of chemokines and cytokines in peritoneal lavage 4 h after PBS or LPS treatment of WT, $CD19^{-/-}$, $BMyD88^{-/-}$ and μMT dams (in pg/ml). Data were analyzed by Kruskal-Wallis-test, followed by Dunn´s multiple comparisons test (* significant compared to WT LPS), shown are the p values from the Kruskal-Wallis test and the mean values, n= 5-6 mice/group.

	WT PBS	WT LPS	CD19$^{-/-}$ PBS	CD19$^{-/-}$ LPS	BMyD88$^{-/-}$ PBS	BMyD88$^{-/-}$ LPS	μMT PBS	μMT LPS	*p*
chemokines									
BLC	1.9	22.6	4.5	26.9	0.2	22.5	21.1	36.0	0.0160
LIX	25.0	96.8	21.7	112.1	50.4	121.9	87.3	168.5*	<0.0001
MDC	44.7	43.2	25.7	87.4	16.7	87.3	58.8	231.4*	0.0094
MIG	3.0	31.1	1.7	54.9	24.4	49.9	5.7	97.6	0.0088
MIP-1α	0	39.6	0	64.4	0	43.4	0	111.7	0.0044
Eotaxin	1.4	0	0	31.7	0	41.1*	0	12.5	0.0074
KC	19.3	68.3	51.6	105.5	34.9	144.9*	36.2	118.6	0.0162
MCP-1	22.3	143.2	20.2	254.0	0	232.5	42.6	290.0	<0.0001
MIP-3α	0	0	0	7.7	0	19.4	0	22.7*	0.0021
RANTES	0	0	0	0	0	0	0	20.8*	0.0004
TARC	0	0	0	63.6	0	92.8*	25.1	105.5*	<0.0001
cytokines									
IL-6	0.3*	17703	1.3*	13659	1.4*	11558	1.7*	17392	<0.0001
TNF-α	5.7*	1370	12.6*	1062	13.0*	978.8	10.6*	1050	<0.0001
IL-2	5.7	0.3	0.2	0.3	0.3	0.2	0.2	0.2	0.9179
IL-4	0.3	0.4	0.4	0.7	0.4	0.4	0.1	0.7	0.2762
IL-10	7.1*	65.9	3.5*	28.3*	6.4*	26.8*	10.5*	32.3*	<0.0001
IFN-γ	3.6	14.4	0.5	6.2	3.1	4.7	0.5	27.4	0.0181
IL-17A	0.4*	8.0	0.7*	2.3	0.6*	2.6	1.5	4.5	0.0020

Table S2: Levels of chemokines and cytokines in maternal serum 4 h after PBS or LPS injection in the peritoneal cavity of WT, CD19$^{-/-}$, BMyD88$^{-/-}$ and μMT dams (in pg/ml). Data were analyzed by Kruskal-Wallis test, followed by Dunn´s multiple comparisons test (* significant compared to WT LPS), shown are the p values from the Kruskal-Wallis test and the mean values, n= 5-6 mice/group.

	WT PBS	WT LPS	CD19$^{-/-}$ PBS	CD19$^{-/-}$ LPS	BMyD88$^{-/-}$ PBS	BMyD88$^{-/-}$ LPS	μMT PBS	μMT LPS	*p*
IFN-γ	25.2	26.7	19.1	29.0	20.1	24.3	23.0	27.7	0.1547
IL-12p70	28.6	101.2	24.1	223.1	25.1	55.6	19.22*	114.2	0.0004
IL-1α	27.4	100.9	173.3	501.9*	29.2	346.1*	30.2	308.1*	<0.0001
IL-23	39.2	45.7	27.1	47.8	40.4	42.5	36.2	51.0	0.0640
MCP-1	24.1	304.6	120.5	527.3	26.5	221.3	11.2	275.6	0.0683
TNF-α	46.2	34.7	77.8	137.7	31.8	144.8	55.7	76.5	0.1476
GM-CSF	20.5	28.3	20.4	26.6	25.7	14.7	33.2	2.1*	0.0138
IFN-β	23.1	26.5	11.2*	24.6	10.8*	21.8	9.9	29.8	0.0072
IL-6	23.7	144.0	213.8	743.9	303.0	580.7	98.2	1133	0.0006
IL-10	20.3*	56.3	24.2*	19.0*	30.4	20.2*	24.9	21.3*	0.0069
IL-17A	16.0	24.2	8.6*	22.6	15.2	21.9	16.4	27.5	0.0307
IL-1β	18.2	16.7	3.8	17.9	5.1	8.3	15.8	38.5	0.0732
IL-27	27.7	37.0	31.6	37.8	29.0	38.5	28.0	38.4	0.0374

Table S3: Concentrations of cytokines placenta supernatant from 4 h PBS or LPS-treated WT, CD19$^{-/-}$, BMyD88$^{-/-}$ and μMT dams (in pg/ml). Data were analyzed by Kruskal-Wallis test, followed by Dunn´s multiple comparisons test (* significant compared to WT LPS), shown are the p values from the Kruskal-Wallis test and the mean values, n= 5-6 mice/group.

	WT PBS	WT LPS	CD19$^{-/-}$ PBS	CD19$^{-/-}$ LPS	BMyD88$^{-/-}$ PBS	BMyD88$^{-/-}$ LPS	μMT PBS	μMT LPS	*p*
amniotic fluid									
IL-6	18.7	21.6	10.3	152.4*	20.4	235.8*	9.7	56.7	<0.0001
TNF-α	1.2	5.8	0.6	18.2	2.8	42.3	0.7	10.5	0.0009
IL-2	0	0.4	0.9	1.1	0.3	0.6	0.2	0.3	0.0203
IL-4	1.1	1.4	1.1	1.8	1.5	1.4	0.7	1.7	0.1720
IL-10	9.4	17.9	15.0	13.0	9.7	8.4*	2.7*	3.6*	0.0005
IFN-γ	0.3	0.1	0.3	0.5	0.5	1.1*	0.1	0.4	0.0046
IL-17A	0.1	0.1	0.2	0.4	0.6	1.0	0.1	0.2	0.0637
fetal serum									
IL-6	6.8	25.9	4.5	2702*	16.9	1732	7.7	3390*	<0.0001
TNF-α	14.5	15.9	11.2	341.8*	15.0	65.9	11.0	70.0	0.0007
IL-2	0.4	0.7	0.7	0.5	0.3	0.3	0.2	0.4	0.5493
IL-4	0.1	1.6	1.9	0.7	0.5	1.0	1.2	0.5	0.3039
IL-10	12.8	16.0	5.1	3.0	1.7*	3.4	5.3	5.1	0.0200
IFN-γ	0.3	0.3	0.9	0.1	0.2	0.7	0.4	1.1	0.2691
IL-17A	0.4	0.2	0.6	0.4	0.4	0.5	1.2	0.4	0.4804

Table S4: Concentrations of cytokines in amniotic fluid and fetal serum from 4 h PBS or LPS-treated WT, CD19$^{-/-}$, BMyD88$^{-/-}$ and μMT dams (in pg/ml). Data were analyzed by Kruskal-Wallis-test, followed by Dunn´s multiple comparisons test (* significant compared to WT LPS), shown are the p values from the Kruskal-Wallis test and the mean values, n= 5-6 mice/group.

	Forward primer	Reverse primer	FAM-labeled probe
Il1b	CAA CCA ACA AGT GAT ATT CTC CAT G	GAT CCA CAC TCT CCA GCT GCA	
Il6	TCC AGA AAC CGC TAT GAA GTT CC	GTC ACC AGC ATC AGT CCC AAG	
Ccl5	GCA AGT GCT CCA ATC TTG CA	GAT GTA TTC TTG AAC CCA CTT CTT CTC	
Mmp9	CAA TCC TTG CAA TGT GGA TG	TAA GGA AGG GGC CCT GTA AT	
Actb	TGC GTC TGG ACC TGG CTG G	ATC CTG TCA GCA ATG CCT GGG	
Il10	GAA GAC CCT CAG GAT GCG G	CCT GCT CCA CTG CCT TGC T	CGC TGT CAT CGA TTT CTC CCC TGT GA
Tnfa	TCG AGT GAC AAG CCC GTA GC	CTC AGC CAC TCC AGC TGC TC	CGT CGT AGC AAA CCA CCA AGC GGA GGA
Ifng	CTC ATG GAG CTG CAG AGA CTC TTT	GTG ATG TGG ACT TGG ACT CAT TCA	ATG CCT GGA TTC ATC GAT AAG CTG CAC CT
Actb	GCT TCT TTG CAG CTC CTT CGT T	GTT GTC GAC GAC CAG CGC	CGC CAC CAG TTC GCC ATG GAT

Table S5: List of used primers.

	WT PBS	WT LPS	CD19-/- PBS	CD19-/- LPS	BMyD88-/- PBS	BMyD88-/- LPS	µMT PBS	µMT LPS	*p*
uterus									
tnfa	0.000362	0.004032	0.000740	0.004670	0.000115*	0.003854	0.000490	0.002350	0.0010
Il10	0.000124*	0.004383	0.000118	0.002600	0.000110	0.003758*	0.000118	0.001768	0.0005
Ifng	0.000270	0.000316	0.000150	0.000583	0.000158	0.000476	0.000255	0.000415	0.0899
Il6	0.000026	0.001633	0.000040	0.001115	0.000015	0.004340	0.000023	0.002182	0.0004
Mmp9	0.000026	0.000058	0.000073	0.000043	0.000055	0.000044	0.000073	0.000075	0.1086
Il1b	0.000196	0.005566	0.000518	0.007108	0.000290	0.008434	0.000165*	0.005518	0.0007
Ccl5	0.003608	0.135900	0.002765*	0.085170	0.005313	0.163400	0.003973	0.194800	0.0003
amnion									
tnfa	0.000642*	0.008078	0.000598	0.015320	0.000326*	0.007546	0.001226	0.003528	0.0001
Il10	0.000362*	0.006032	0.000114*	0.012980	0.000124*	0.009524	0.000312	0.002288	0.0003
Ifng	0.000240	0.000498	0.000252	0.000364	0.000184	0.000768	0.000230	0.000215	0.1981
Il6	0.000020	0.000763	0.000040	0.003318	0.000036	0.002368	0.000010	0.001808	0.0002
Mmp9	0.000008	0.000003	0.000022	0.000010	0.000578	0.000005	0.000012	0	0.3758
Il1b	0.000076	0.005898	0.000128	0.010020	0.000530	0.011070	0.000224	0.005732	<0.0001
Ccl5	0.017050	0.267000	0.035560	0.525300	0.028160	1.732000	0.032110	1.193000	0.0017
placenta									
tnfa	0.000803*	0.002473	0.000546*	0.004230	0.001058	0.002108	0.001092	0.002720	0.0055
Il10	0.000030	0.000047	0.000014	0.000035	0.000028	0.000037	0.000014	0.000018	0.7688
Ifng	0.000052	0.000022	0.000020	0.000028	0.000020	0.000018	0.000104	0.000042	0.0766
Il6	0.000224	0.000535	0.000040	0.002305	0.000115	0.001306	0.000052	0.003428*	<0.0001
Mmp9	0.000110	0.000122	0.000086	0.000286	0.000028	0.000108	0.000052	0.000180	0.1077
Il1b	0.001376*	0.012040	0.000560*	0.003800	0.003246	0.005185	0.001266*	0.007736	0.0001
Ccl5	0.005410	0.015960	0.004063	0.021530	0.012180	0.019470	0.002210*	0.040000	0.0008

Table S6: Expression of *TNF-α, IL-10, IFN-γ, IL-6, MMP9, IL-1β* and *RANTES* mRNA in uterus, amnion and placenta 4 h after PBS or LPS treatment. Data were analyzed by Kruskal-Wallis-test, followed by Dunn´s multiple comparisons test (* significant compared to WT LPS), shown are the p values from the Kruskal-Wallis-test and the mean values, n= 5-6 mice/group.

	WT PBS	WT LPS	$CD19^{-/-}$ PBS	$CD19^{-/-}$ LPS	$BMyD88^{-/-}$ PBS	$BMyD88^{-/-}$ LPS	µMT PBS	µMT LPS	*p*
fetal liver									
tnfa	0.000707	0.000666	0.000614	0.000384	0.000794	0.001170	0.000966	0.000848	0.0204
Ifng	0.000017	0.000010	0.000030	0.000248*	0.000010	0.000013	0.000064	0.000078*	0.0009
Il6	0.000172	0.000568	0.000034*	0.002188	0.000260	0.000350	0.000052*	0.000108	<0.0001
Mmp9	0.000385	0.000397	0.000055*	0.000770*	0.000234	0.000335	0.000088	0.000100	<0.0001
Il1b	0.000750	0.004153	0.000698	0.010850	0.000200	0.000837	0.001696	0.003044	0.0125
Ccl5	0.011820	0.015740	0.016630	0.024310	0.006740	0.004176	0.033090	0.01349	0.1455
fetal lung									
tnfa	0.000124	0.000249	0.000314	0.000256	0.000252	0.000713	0.000130	0.000083	0.0125
Ifng	0.000300	0.000098	0.000284	0.000120	0.000078	0.000053	0.000240	0.000050	0.0475
Il6	0.000385*	0.000003	0.000009	0.000038	0.000014	0.000020	0.000020	0.000512	0.0076
Mmp9	0.000023	0.000041	0.000077	0.000060	0.000132	0.000102	0.000032	0.000267	0.6535
Il1b	0.000067	0.000409	0.000552*	0.001010*	0.000136	0.000755	0.000660	0.001752*	0.0016
Ccl5	0.002417	0.001391	0.023460*	0.002297	0.011720	0.007608	0.013960	0.002440*	0.0097
fetal gut									
tnfa	0.000358	0.000408	0.000556	0.000088	0.000280	0.000298	0.000916	0.000270	0.0225
Ifng	0.000044	0.000056	0.000055	0.000038	0.000040	0.000058	0.000066	0.000044	0.6612
Il6	0.000013	0.000078	0.000022	0	0.000012	0.000002	0.000036	0.000008	0.0922
Mmp9	0.000178	0.000060	0.000217	0.000212	0.000192	0.000124	0.000048	0.000058	0.0792
Il1b	0.000372	0.001110	0.000222	0.000226	0.000240	0.000208	0.000214	0.000216	0.0257
Ccl5	0.111600	0.225600	0.011160	0.005998	0.007070	0.003885*	0.005902	0.004296*	0.0008
fetal brain									
tnfa	0.000012	0.000008	0.000012	0.000020	0.000010	0.000015	0.000008	0.000025	0.0623
Ifng	0	0.000005	0.000002	0.000008	0.000003	0.000005	0.000004	0.000013	0.1995
Il6	0.000007*	0.000083	0.000002*	0.000003*	0.000007*	0.000005*	0.000010	0.000005*	0.0042
Mmp9	0.000242	0.000140	0.000703*	0.000275	0.000175	0.000395	0.000154	0.000495	0.0009
Il1b	0.000050	0.000136	0.000022	0.000728	0.000058	0.000040	0.000036	0.000598	0.1344
Ccl5	0.000048	0.000327	0.000125	0.000045	0.000078	0.000038	0.000028	0.000103	0.6890

Table S7: Expression of *TNF-α, IFN-γ, IL-6, MMP9, IL-1β* and *RANTES* mRNA in fetal liver, lung, gut and brain 4 h following PBS or LPS injection. Data were analyzed by Kruskal-Wallis test, followed by Dunn´s multiple comparisons test (* significant compared to WT LPS), shown are the p values from the Kruskal-Wallis test and the mean values, n= 5-6 mice/group.
